# Investigating ^18^F-FDG PET/CT Parameters as Prognostic Markers for Differentiated Thyroid Cancer: A Systematic Review

**DOI:** 10.3389/fonc.2021.648658

**Published:** 2021-05-13

**Authors:** Hongxi Wang, Hongyuan Dai, Qianrui Li, Guohua Shen, Lei Shi, Rong Tian

**Affiliations:** ^1^Department of Nuclear Medicine, West China Hospital, Sichuan University, Chengdu, China; ^2^Department of Nuclear Medicine, Chengdu Fifth People's Hospital, Chengdu, China

**Keywords:** ^18^F-FDG PET/CT, differentiated thyroid carcinoma, outcome, systematic review, prediction

## Abstract

**Aims:** The aim of this study was to determine whether ^18^F-fluorodeoxyglucose positron emission tomography/computed tomography (^18^F-FDG PET/CT) parameters might be prognostic markers for patients with differentiated thyroid carcinoma (DTC).

**Methods:** We searched for eligible articles in PubMed, EMBASE (Ovid), Cochrane Library, and ClinicalTrials.gov from inception to February 2021. We included studies addressing the association between ^18^F-FDG PET/CT parameters and clinical outcomes among patients with DTC. Quality assessment was performed using the Quality in Prognosis Studies (QUIPS) tool.

**Results:** A total of 25 studies including 2,954 patients (1,994 females, 67.5%) were included; 2,416 patients (81.8%) had papillary thyroid carcinoma (PTC), and the mean or median follow-up time ranged from 19.1 months to 17.1 years. Thirteen (52.0%) studies were assessed as “unclear” for the domain of study participation. The most common timing of PET/CT scans was after thyroidectomy (in 20 of 25 studies, 80%), especially in patients with an elevated thyroglobulin (Tg) and a negative radioiodine whole-body scan (WBS). The most common PET parameter was FDG uptake. Twelve of 17 (70.6%) and 12 of 12 (100%) studies showed an association between PET/CT parameters and disease progression and survival in patients with DTC, respectively.

**Conclusion:** ^18^F-FDG PET/CT parameters alone or combined with other variables can serve as prognostic markers to identify DTC patients with poor outcomes, especially in the setting of an elevated Tg and a negative WBS. Future research is needed to confirm these findings and to examine the prognostic value of PET/CT parameters for DTC patients, considering the heterogeneity in PET/CT parameters, unclear information of patients, and PET/CT-adapted treatment modifications.

## Introduction

Differentiated thyroid carcinoma (DTC) is the most common endocrine tumor with an increasing incidence worldwide. DTC has a generally good prognosis, with an overall mortality rate of <10% (1, 2). However, ~10–30% of DTC patients develop metastatic or recurrent diseases, among whom 33–50% eventually progress into radioiodine-refractory (RAI-R) diseases (1, 2). The identification of predictors of clinical outcomes for DTC patients is of immense clinical value (3, 4).

^18^F-fluorodeoxyglucose positron emission tomography/computed tomography (^18^F-FDG PET/CT), combining functional and anatomic information, has become a valuable tool for the staging, treatment response assessment, prognosis prediction, and surveillance of patients with various malignancies (5). The American Thyroid Association (ATA) and the National Comprehensive Cancer Network (NCCN) guidelines have recommended that PET/CT should be considered for detecting metastasis or recurrence in patients with elevated thyroglobulin (Tg) and negative whole-body scans (WBS) during follow-up (6, 7). Recently, it has been widely illustrated that PET/CT parameters at different times are associated with established prognostic variables, such as age, the level of Tg, tumor size, BRAF mutation, etc. (8). Thus, PET/CT may provide additional prognostic information compared with clinical prognostic variables for DTC patients. Although the diagnostic and staging value of PET/CT in DTC patients has been examined in several studies (9, 10), limited data are available to evaluate the potential of PET/CT parameters as prognostic variables in DTC patients (11).

Therefore, the aim of this systematic review was to report the available evidence on the value of ^18^F-FDG PET/CT parameters to predict outcomes in patients with DTC.

## Materials and Methods

This systematic review was performed according to the PRISMA statement (12). The PRISMA checklist is provided in Supplementary Table 1.

### Eligibility Criteria

We included retrospective or prospective cohort studies assessing ^18^F-FDG PET or PET/CT parameters as prognostic factors in univariate or multivariate analyses to predict outcomes in DTC patients. At least 10 patients were involved and sufficient survival data, including overall/progression/recurrence/disease/event-free survival (OS/PFS/RFS/DFS/EFS, respectively), were reported.

### Search Strategy

We performed a comprehensive literature search to identify English language studies published in the PubMed, EMBASE (Ovid), Cochrane Library, and ClinicalTrials.gov from inception to March 2020. We used the following search strategy: (thyroid carcinoma OR thyroid cancer) AND (PET OR positron emission tomography OR FDG) AND (Prognos^*^ OR survival OR outcome). The references cited in the retrieved studies were also explored to include potentially eligible studies.

### Literature Screening and Data Extraction

Two reviewers independently screened titles, abstracts, and full texts for eligibility and extracted the following information from each included study: (1) general information of the study (author, publication year, country, and study type); (2) patient characteristics and clinical outcomes (sample size, age, gender, histology, treatment, outcomes, and follow-up); and (3) prediction results/prediction efficiency (univariate and multivariate analysis results).

### Quality Assessment

The quality of the studies was independently assessed by two reviewers using the Quality in Prognosis Studies (QUIPS) tool (Supplementary Table 2) (13). Any disagreement was resolved through discussion with a third reviewer.

## Results

### Literature Selection and Quality Assessment

**Figure 1:**
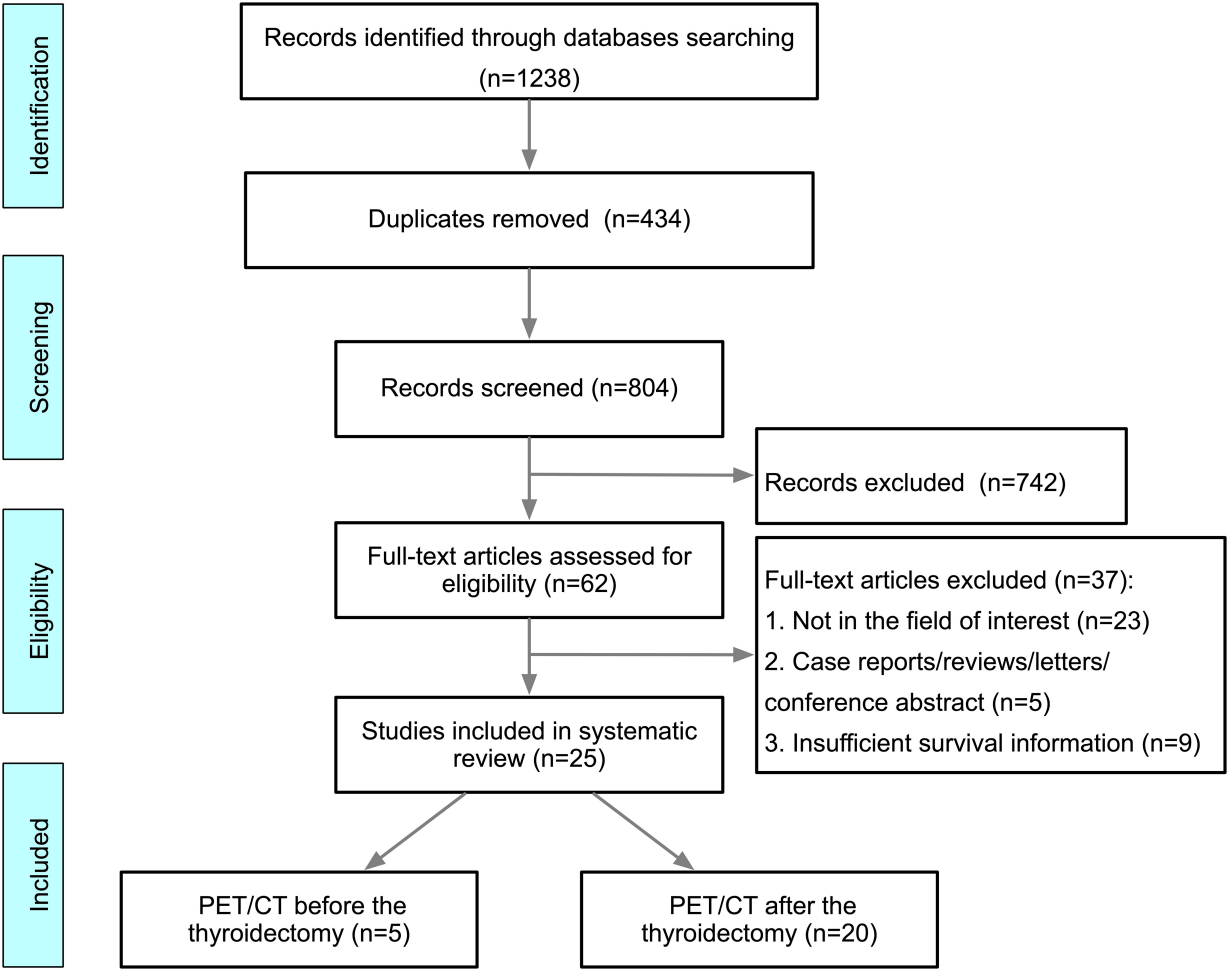
Flow diagram of the studies included in the current systematic review.

**Figure 2:**
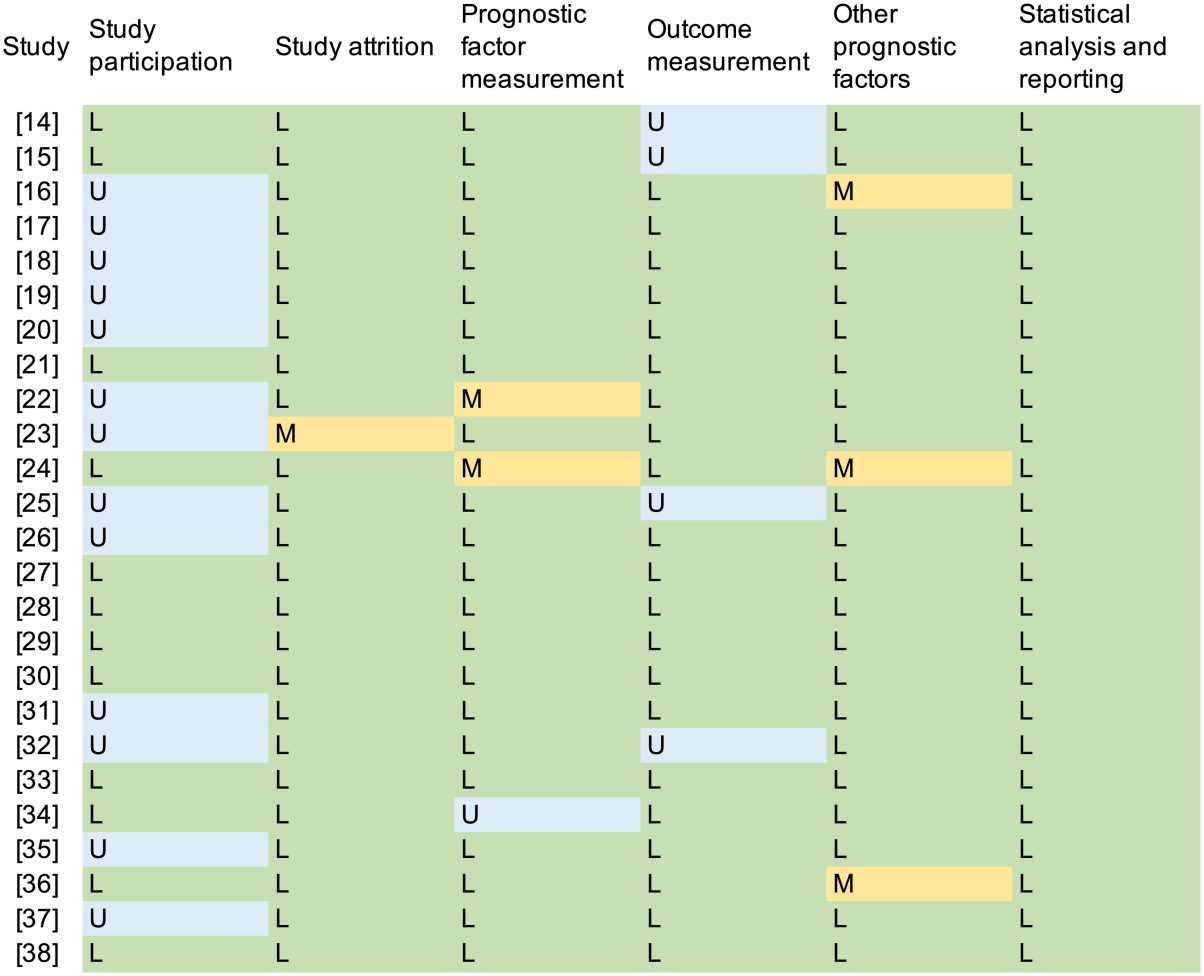
Quality assessment according to the QUIPS. *L*, low risk; *M*, moderate risk; *H*, high risk; *U*, unclear.

A total of 1,238 papers were found and the full texts of 62 papers were screened. Among these articles, 37 studies were excluded. Ultimately, 25 studies (14–38) were included in this systematic review (Figure 1). Thirteen (52.0%) studies (16–20, 22, 23, 25, 26, 31, 32, 35, 37) were assessed as “unclear” for the domain of study participation, mostly due to a lack of information about the source population and the population of interest (TNM stage, histology, etc.), or ambiguous inclusion and exclusion criteria. The risk of bias for outcome measurement was assessed as “unclear” in four studies (14, 15, 25, 32) due to a lack of outcome definition. Three studies (16, 24, 36) were assessed to have a “moderate” risk of bias in the domain of other prognostic factors (covariates) because they did not consider other clinical variables (Figure 2).

### Characteristics of the Studies

Table 1 shows the characteristics of the included studies. Six, 12, six, and one studies assessed European, Asian, North American, and South American populations, respectively. The study periods ranged from 1983 to 2018, and 21 studies (84%) were developed before 2015. The sample sizes ranged from 17 to 412. A total of 2,954 patients (1,994 females, 67.5%) were included. Their ages ranged from 8 to 89 years; 2,416 (81.8%) and 227 (7.6%) patients had papillary thyroid carcinoma (PTC) and follicular thyroid cancer (FTC), respectively. The most common PET/CT parameter was fluorodeoxyglucose (FDG) uptake (in 16 studies). The mean or median follow-up time ranged from 19.1 months to 17.1 years. The end point was overall survival (OS) in 12 studies and PFS/RFS/DFS/EFS in 17 studies. The results of the included studies are shown in Table 2.

**Table 1:** Summary of characteristics of the included patients.

**References**	**No. of patients** ** (female)**	**Age (years)^a^**	**Histology**	**T (T1, T2, T3, T4)**	**N (N0, N1)**	**M (M0, M1)**	**Stage**	**Follow-up^a^**
**Before thyroidectomy**								
Kwon et al. (14)	274 (228)	48 (13–77)	PTC	131, 7, 109, 27	86, 188	274, 0	NR	37.8 ± 13.9 months (12–76)
Lee et al. (15)	96 (72)	44.5	PTC	13, 3, 69, 11	0, 96	96, 0	NR	50 months
Kim et al. (16)	197 (151)	50.2 (15–83)	PTC	NR	0, 90	NR	NR	6–46 months
Kim et al. (17)	412 (340)	47.2 ± 12.2 (17–84)	PTC	NR	214, 161	412, 0	NR	43.9 ± 16.6 months (1.9–87.0)
Qiu et al. (18)	80 (51)	53 (17–81)	PTC (41), FTC (39)	NR	NR	0, 80	NR	3.37 years (1–4.93)
**After thyroidectomy**								
Pryma et al. (19)	44 (21)	62 (24–81)	Hürthle cell	NR	NR	NR	NR	2.9 years (1.2–8.8)
Nagamachi et al. (20)	70 (48)	55.2 ± 23	PTC (62), FTC (8)	NR	NR	NR	I–III 39, IV 31	4.6 ± 0.6 years
Pace et al. (21)	60 (48)	44 ± 14 (18–79)	PTC (51), FTC (9)	NR	NR	57, 3	I 44, II 13, III 3	31.7 ± 20.6 months (6–67)
Salvatore et al. (22)	83 (58)	44.1 ± 17.1	PTC (76), FTC (7)	NR	NR	0, 83	NR	111.9 ± 91.6 months (15–159)
Zhu et al. (23)	141 (88)	58.6 ± 14.2	PTC (127), FTC (14)	NR	NR	0, 125	NR	54.1 ± 33.0 months (6.3–124.1)
Gaertner et al. (24)	125 (81)	48.2 (7–81)	PTC (93), FTC (18), Hürthle cell (12), anaplastic (2)	6, 20, 12, 69	26, 77	67, 58	I 30, II 21, III 27, IV 47	NR
Wang et al. (25)	49 (29)	54.3 ± 17.4	PTC (31), FTC (18)	NR	NR	32, 17	NR	7.9 ± 5 years (1–20)
Robbins et al. (26)	400 (225)	53.8 ± 16.1	PTC (277), FTC (31), Hürthle cell (36), poorly differentiated (45), anaplastic (11)	NR	NR	NR	I 139, II 56, III 133, IV 62	7.9 years (0.15–39.7)
Deandreis et al. (27)	80 (46)	55 ± 19	PTC (45), FTC (12), Other (23)	8, 8, 13, 24	14, 40	0, 80	NR	4.2 ± 4.3 years
Hong et al. (28)	64 (47)	49.9 ± 16.4	PTC (52), FTC (12)	0, 5, 42, 3	14, 43	0, 64	NR	38.5 months (1–79)
Akkas et al. (29)	77 (45)	53.7 ± 15 (19–83)	PTC (64), FTC (6), Hürthle cell (7)	NR	NR	0, 77	I 23, II 9, III 15, IV 30	4.8 ± 1.3 years
Masson-Deshayes et al. (30)	37 (26)	61.8 ± 13.3	NR	5, 4, 18, 8	16, 13	0, 37	NR	3.5 years
Marcus et al. (31)	202 (125)	NR	PTC (184), FTC (18)	NR	NR	NR	I 68, II 8, III 36, IV 26	94 months (6.17–534.1)
Manohar et al. (32)	62 (25)	63.2 ± 13.1 (16–89)	PTC (44), FTC (4), other (14)	NR	NR	NR	I 3, II 3, III 18, IV 38	11.1 years (1.2–20)
Pitoia et al. (33)	24 (17)	NR	PTC (18), FTC (6)	NR	NR	0, 24	II 13, IV 11	17.1 ± 1.4 years (3–21)
Sabra et al. (34)	199 (105)	50 ± 20	PTC (119), Hürthle cell (14), poorly differentiated (66)	NR	NR	0, 115	I 33, II 40, III 18, IV 102	6.9 years
Kang et al. (35)	66 (47)	48.5 ± 15.5 (25–77)	PTC	NR	NR	NR	NR	30.5 ± 17.2 months (12–93)
Wang et al. (36)	20 (10)	54.7 ± 13.3 (27–78)	PTC (18), FTC (2)	2, 0, 4, 10	1, 15	0, 20	NR	3.7–17.53 months
Kim et al. (37)	85 (52)	55 (22–81)	PTC (60), FTC (17), poorly differentiated (8)	NR	NR	0, 82	NR	19.1 months (1.8–92.2)
Marotta et al. (38)	17 (9)	61	PTC (7), FTC (10)	NR	NR	0, 17	II 1, III 8, IV 8	NR

*PTC, papillary thyroid cancer; FTC, follicular thyroid cancer; NR, not reported*.

a *Mean ± SD/median (range)*.

**Table 2:** Summary of the main results of the included studies.

**References**	**PET/CT parameters**	**Timing and indication of PET/CT**	**End point**	**Univariate analysis^a^**	**Multivariate analysis^a^**
**Before thyroidectomy**					
Kwon et al. (14)	Tumor-to-liver uptake ratio (TLR)	Within 3 months of surgery	DFS	NR	NS
Lee et al. (15)	SUV_max_ of metastatic lymph nodes	Before surgery	RFS	*p* = 0.025	NR
Kim et al. (16)	FDG uptake of primary tumor	Before surgery	RFS	NS	NR
	FDG uptake of lateral neck node metastasis		RFS	NS	NR
Kim et al. (17)	FDG uptake of primary tumor	Within 3 months prior to surgery	DFS	*p* = 0.0049	NS
Qiu et al. (18)	FDG uptake of bone lesions	Before thyroidectomy or after 131-I therapy	OS	*p* = 0.013	HR = 4.13 (95% CI = 3.96–4.27), *p* = 0.009
**After thyroidectomy**					
Pryma et al. (19)	SUV_max_	After thyroidectomy. An elevated Tg, abnormal imaging, high-risk histopathology	OS	*p* < 0.01	NR
Nagamachi et al. (20)	FDG uptake	Before 131-I therapy	OS	*p* < 0.05	RR = 5.01 (95% CI = 3.41–6.62), *p* < 0.011
Pace et al. (21)	FDG uptake	Before 131-I therapy	DFS	*p* = 0.001	χ^2^ = 16.1, HR = 5.5, *p* < 0.0005
Salvatore et al. (22)	FDG uptake	Before or after 131-I therapy	PFS	*p* = 0.000	NR
Zhu et al. (23)	FDG uptake	Before remnant ablation. In setting of suspicion or proven metastases	OS	*p* < 0.05	NR
Gaertner et al. (24)	FDG uptake, SUV_max_, volume of lesions	After 131-I therapy. A negative DxWBS with elevated Tg, high risk, known distant metastases	OS	*p* = 0.001	*p* < 0.05
Wang et al. (25)	FDG uptake	After remnant ablation, an elevated Tg	DFS	*p* < 0.001	χ^2^ = 26.3, *p* < 0.0001
			OS	*p* < 0.05	NS
Robbins et al. (26)	FDG uptake, number of lesions, SUV_max_	An elevated Tg with negative WBS, surveillance in Hürthle cell carcinoma	OS	*p* < 0.001	RR = 7.69 (95% CI = 2.17–24.4), *p* < 0.05
Deandreis et al. (27)	FDG uptake, SUV_max_, the number of lesions	At the time of diagnosis or during follow-up. To detect or assess metastases	PFS	*p* = 0.01	NS
			OS	*p* = 0.009	*p* = 0.001
Hong et al. (28)	FDG uptake, SUV_max_	The interval between PET/CT and RxWBS was <12 months.	DSS	*p* < 0.001	HR = 10.53 (95% CI = 1.95–56.75), *p* = 0.006
Akkas et al. (29)	Location of lesions, number of lesions, SUV_max_	After I-131 treatment in recurrent DTC. An elevated Tg with a negative RxWBS or a positive RxWBS with an elevated Tg	DSS	NS	NR
Masson-Deshayes et al. (30)	SUV_max_, SUL_peak_, MTV, TLG, number of lesions	After the diagnosis of distant metastases	PFS	HR = 3.96 (95% CI = 1.76–8.89), *p* = 0.001	*p* < 0.05
			OS	HR = 4.41 (95% CI = 1.39–14.01), *p* = 0.012	NR
Marcus et al. (31)	FDG uptake	After I-131 treatment. An elevated Tg and a negative WBS or at the time of suspected recurrence	OS	HR = 6.1 (95% CI = 3.0–14.3), *p* < 0.0001	*p* < 0.0001
Manohar et al. (32)	MTV, TLG	After 131-I therapy. An elevated Tg with a negative WBS	DFS	HR = 1.21 (95% CI = 1.05–1.39), *p* = 0.005	NR
			OS	HR = 1.17 (95% CI = 0.99–1.39), *p* = 0.05	NR
Pitoia et al. (33)	FDG uptake	After remnant ablation	OS	*p* = 0.0003	HR = 9.11 (95% CI = 0.99–32.22), *p* = 0.0003
Sabra et al. (34)	FDG uptake	NR	PFS	*p* < 0.0001	NR
Kang et al. (35)	SUV_max_	Within 6 months before surgery for recurrent PTC. As preoperative workup	DFS	*p* < 0.001	NR
Wang et al. (36)	ΔSUV_max_%, ΔMTV%, ΔTLG%	In the setting of the apatinib treatment	PFS	*p* = 0.0001	NR
Kim et al. (37)	FDG uptake	In the setting of the sorafenib treatment	PFS	NS	NR
Marotta et al. (38)	Baseline SUV_max_, reductions in SUV_max_	In the setting of the sorafenib treatment	PFS	NS	NR

*NR, not reported; NS, not significant; HR, hazard ratio; RR, relative risk; 95% CI, 95% confidence interval*.

a *Univariate analysis was performed using Kaplan–Meier survival plots and the log-rank test or the Cox regression model. Multivariate analysis was performed using the Cox regression model or Cox proportional hazards model. All effect values are the highest values in the studies*.

### PET/CT Before Thyroidectomy

Five studies (14–18) investigated the prognostic value of PET/CT parameters in patients with DTC before thyroidectomy. Three studies (15, 17, 18) suggested the potential prognostic value of PET/CT parameters in this setting. In a study of DTC patients with bone metastases (18), FDG uptake of bone lesions was an independent predictor of OS [hazard ratio (HR) = 4.13, 95% CI = 3.96–4.27, *p* = 0.009] according to multivariate analysis. In contrast, two studies did not find associations between the tumor-to-liver uptake ratio (TLR) and disease-free survival (DFS) (14) or between the FDG uptake of primary lesion/lateral neck node metastasis and recurrence-free survival (RFS) (16).

### PET/CT After Thyroidectomy

Twenty studies (19–38) explored the association between the PET/CT parameters after thyroidectomy and the outcomes of DTC patients. The common indications of PET/CT before radioactive iodine (131-I) therapy included an elevated Tg, abnormal imaging (WBS, US, and CT), high-risk histopathology, and suspicion or proven metastases. Four studies did not report the indication (20–22, 34).

For DFS/PFS/disease-specific survival (DSS), 10 studies (21–23, 27, 28, 30, 32, 34–36) reported associations between the PET/CT parameters and DFS/PFS/DSS using univariate analysis. Five (21, 25, 27, 28, 30) studies further performed a multivariate analysis, four of which (21, 25, 28, 30) reported that the FDG uptake, maximum standardized uptake value (SUV_max_), peak standardized uptake value corrected for lean body mass (SUL_peak_), and number of lesions were associated with DFS/DSS. In contrast, three studies (29, 37, 38) reported no association between the location of FDG-avid lesions, number of FDG-avid lesions, SUV_max_ (29), FDG uptake (37), baseline SUV_max_ or reductions in SUV_max_ of lesions (38), and disease progression.

Eleven studies (19, 20, 23–27, 30–33) explored whether the PET/CT parameters were associated with the survival of DTC patients, and all found an association in univariate analysis. Seven studies (20, 24–27, 31, 33) performed a multivariate analysis, and FDG uptake (20, 26, 27, 31, 33), volume of lesions (24), number of lesions (26), and SUV_max_ (26) were associated with OS, with a higher predictive value than age (24, 26, 33), sex (24, 33), or metastasis status (24, 26) alone. Only one study (20) reported that FDG uptake of lesions was not a significant predictor of survival in multivariate analysis.

## Discussion

This is the first systematic review about the prognostic value of ^18^F-FDG PET/CT parameters for the clinical outcomes of patients with DTC. Most studies suggested PET/CT parameters as promising prognostic markers: 12 of 17 (70.6%) and 12 of 12 (100%) studies showed an association between the PET/CT parameters and disease progression and survival in patients with DTC, respectively. However, the potential confounders caused by the heterogeneity in PET/CT parameters, unclear information on patients, and PET/CT-adapted treatment modifications should be considered. The prognostic value of ^18^F-FDG PET/CT in DTC is not yet generalizable and should be explained with caution.

Primarily, the role of PET/CT has been limited to detecting lesions responsible for elevated Tg in patients with a negative WBS or to determining disease extent in patients with elevated Tg along with positive WBS (6, 7). We found that the PET/CT parameters in this clinical setting can provide additional prognostic information as well. For instance, Pace et al. (21) found that patients with negative FDG uptake had a better progression-free survival (PFS) either in the whole group or in those with elevated Tg (both >2 and >10 ng/ml); only Tg and FDG uptake were independent predictors of PFS in DTC patients. In patients with lung metastasis (31), extrathyroidal invasion, FDG-avid lesions, and metachronous diagnosis of metastasis were independent predictors of OS; age, sex, the moment of diagnosis of lung metastasis, tumor diameter, and the RAI cumulative doses were not. The combination of RAI and FDG uptake was supposed to identify patients with poorer outcomes (24, 26–28), and FDG positivity seems to have a larger influence on prognosis than does RAI uptake (24, 26–28). In a cohort of 64 patients, reduced DSS was observed in patients with FDG (+)/RAI (–) metastatic lesions compared with the FDG (+)/RAI (+) and FDG (–)/RAI (+) groups (28). Deandreis et al. (27) reported that the 2-year survival rates were 60% for PET-positive and 100% for PET-negative patients with metastatic DTC, with no difference between RAI (–)/FDG (+) and RAI (+)/FDG (+) patients. Several studies also reported similar results (24, 26).

Recently, tyrosine kinase inhibitor (TKI) therapy for RAI-R DTC has become a hot topic. The survival of RAI-R DTC was poor, and a study with a median follow-up of 11.1 years (32) reported that, after the diagnosis of metastatic RAI-R disease, the 5-year OS probability of patients was 34%, and the median OS was 3.56 years. The 5-year PFS probability was 19%, and the median PFS was 1.31 years. Not all patients benefit from TKI therapy, and the early identification of subjects with poor response and prognosis is considerably meaningful. A few small-sample studies have explored whether PET/CT parameters can be used as predictors, and the results are controversial (32, 36–38). In a cohort of 20 RAI-R DTC patients treated with apatinib (36), a significant difference between patients with partial response (PR) and stable disease (SD) was observed with respect to ΔMTV% and ΔTLG%; a significant difference in PFS was observed between patients with ΔMTV% at one and two cycles (less than −45% and −45% or greater) and between patients with ΔTLG% at one and two cycles (less than −80% and −80% or greater). In patients who underwent sorafenib therapy (37), the RAI (+) or FDG (+) in lesions did not affect PFS, while larger target lesions (>1.5 cm) and the shortest tumor doubling time (≤6 months) had worse outcomes. Another study (38) reported that baseline SUV_max_ and early reductions in SUV_max_ were higher and more robust in patients who showed disease progression than in patients who responded to sorafenib, but no significant association with PFS was found.

Preoperative PET/CT is not a routine modality in DTC because the incidence of distant metastasis is very low, and a high FDG uptake in tumors makes it difficult to detect adjacent metastatic lymph nodes (39, 40). According to current evidence (14, 16, 17), the FDG uptake of primary tumors before thyroidectomy could not predict disease progression or recurrence, although FDG avidity was more common in patients with confirmed prognostic factors, such as larger tumor size, extrathyroidal extension, and high Tg levels (14, 16, 17). FDG uptake in metastatic lesions before thyroidectomy was associated with poor outcomes; for instance, an SUV_max_ >2.3 of the N1b lymph node was associated with shorter RFS (*p* = 0.025) among 96 PTC patients (15). The FDG uptake of bone lesions was an independent predictor of OS (HR = 4.13, 95% CI = 3.96–4.27, *p* = 0.009) (18).

The most common PET/CT parameter was FDG uptake, visually identifiable FDG activity with a higher intensity than the surrounding tissues and no normal or physiological uptake was considered to be positive. Semiquantitative parameters, such as SUV_max_, metabolic tumor volume (MTV), and total lesion glycolysis (TLG), have also been described in some studies. We noticed considerable differences in the cutoff values of semiquantitative parameters among studies; the cutoff values of SUV_max_ were 10 (19, 24, 27, 30, 35), 2.9 in N1b lymph nodes (16), and 3.6 in distant metastatic lesions (28). The cutoff values of MTV were 9.08 ml (32), 10 and 50 ml (30), and 125 ml (24). The cutoff values of TLG were 49.1 (32) and 154 (30). The different study populations, target lesions, or cutoff measurements (based on previous studies, median values, receiver operating characteristic curves, or log-rank test results) may have led to this difference. Additionally, the semiquantitative parameters did not present higher prognostic values than the conventional parameters in the studies. Masson-Deshayes et al. (30) evaluated the PET/CT scans of 37 patients with metastatic DTC. In the univariate analysis, the prognostic factors for PFS and OS were SUV_max_, SUL_peak_, and TLG. The number of FDG-avid lesions was significantly associated with PFS, but not MTV. The number of FDG-avid lesions and the SUL_peak_ were independent prognostic factors in the multivariate analysis. Dichotomizing patients into two groups of risk could introduce measurement errors and reduce the ability to detect a correlation; keeping variables continuous with linear regression may be relevant (41).

One point raises concern that the effect of PET may be misestimated considering the favorable outcomes attributed to PET/CT-adapted treatment modifications (e.g., dose modification of 131-I, targeted therapy) (42). A retrospective analysis of the likely impact of PET/CT on treatment may be biased. For instance, in 77 patients with recurrent/metastatic DTC (29), lesional SUV_max_, the number or location of FDG-avid lesions, and the TNM stage did not correlate with DSS. This study did not include non-FDG-avid recurrent tumors, and the presence of surgically amenable recurrence/metastasis was considered as a predictor. The prognostic value of PET/CT might be confounded by the type of treatment that is known to be associated with the prognosis. Only one study (25) stated that the results of PET/CT before 131-I therapy did not have any impact on the treatment decision of the patients; they found that FDG uptake (χ^2^ = 26.3, *p* < 0.0001) and Tg were independent predictors of DFS, while Tg was the only variable associated with OS.

This systematic review had some limitations. Firstly, only published English language articles were included, which may lead to publication bias. Secondly, all studies included were retrospective, and, as discussed above, a retrospective analysis of the likely impact of PET/CT on treatment may be biased. Thirdly, we did not contact the authors of the included studies to acquire detailed information of patients. Lastly, we did not perform a cost-effectiveness analysis.

## Conclusion

Current evidence suggests that ^18^F-FDG PET/CT parameters alone or in combination with other variables can serve as prognostic markers to identify DTC patients with poor outcomes, especially when Tg is elevated with a negative WBS. The heterogeneity in PET/CT parameters, unclear information on patients, and PET/CT-adapted treatment modifications may cause potential bias and influence the repeatability of the results. Therefore, larger randomized and prospective research is needed to confirm these findings and to examine the effectiveness of PET/CT parameters at different timings for prognosis assessment in DTC patients. The datasets generated for this study are available on request from the corresponding author.

## Data Availability Statement

The datasets generated for this study are available on request to the corresponding author.

## Author Contributions

Conceptualization: RT, HW, and LS. Investigation: HW and HD. Methodology and Validation: GS and QL. Project administration: HW and RT. Supervision: RT and GS. Visualization: RT and LS. Writing—original draft: HW and HD. Writing—review and editing: GS and RT. All authors contributed to the article and approved the submitted version.

## Conflict of Interest

The authors declare that the research was conducted in the absence of any commercial or financial relationships that could be construed as a potential conflict of interest.
