# Supplementary material for: Investigating 18F-FDG PET/CT Parameters as Prognostic Markers for Differentiated Thyroid Cancer: A Systematic Review
Source: Front Oncol. 2021 May 13;11:648658. doi: 10.3389/fonc.2021.648658 (PMC8158293; doi:10.3389/fonc.2021.648658)
Supplement: Supplementary file 1 [file Data_Sheet_1.docx]

Supplementary Material

# Supplementary Table 1. The PRISMA 2009 Checklist

| **Section/topic** | **#** | **Checklist item** | | **Reported on page #** |  |
| --- | --- | --- | --- | --- | --- |
| **TITLE** | | | |  | |
| Title | 1 | Identify the report as a systematic review, meta-analysis, or both. | | 1 |  |
| **ABSTRACT** | | | |  | |
| Structured summary | 2 | Provide a structured summary including, as applicable: background; objectives; data sources; study eligibility criteria, participants, and interventions; study appraisal and synthesis methods; results; limitations; conclusions and implications of key findings; systematic review registration number. | | 2 |  |
| **INTRODUCTION** | | | |  | |
| Rationale | 3 | Describe the rationale for the review in the context of what is already known. | | 2 |  |
| Objectives | 4 | Provide an explicit statement of questions being addressed with reference to participants, interventions, comparisons, outcomes, and study design (PICOS). | | 3 |  |
| **METHODS** | | | |  | |
| Protocol and registration | 5 | Indicate if a review protocol exists, if and where it can be accessed (e.g., Web address), and, if available, provide registration information including registration number. | | n.a. |  |
| Eligibility criteria | 6 | Specify study characteristics (e.g., PICOS, length of follow-up) and report characteristics (e.g., years considered, language, publication status) used as criteria for eligibility, giving rationale. | | 3 |  |
| Information sources | 7 | Describe all information sources (e.g., databases with dates of coverage, contact with study authors to identify additional studies) in the search and date last searched. | | 3 |  |
| Search | 8 | Present full electronic search strategy for at least one database, including any limits used, such that it could be repeated. | | 3 |  |
| Study selection | 9 | State the process for selecting studies (i.e., screening, eligibility, included in systematic review, and, if applicable, included in the meta-analysis). | | 3 |  |
| Data collection process | 10 | Describe method of data extraction from reports (e.g., piloted forms, independently, in duplicate) and any processes for obtaining and confirming data from investigators. | | 3 |  |
| Data items | 11 | List and define all variables for which data were sought (e.g., PICOS, funding sources) and any assumptions and simplifications made. | | 3 |  |
| Risk of bias in individual studies | 12 | Describe methods used for assessing risk of bias of individual studies (including specification of whether this was done at the study or outcome level), and how this information is to be used in any data synthesis. | | 3 |  |
| Summary measures | 13 | State the principal summary measures (e.g., risk ratio, difference in means). | | n.a. |  |
| Synthesis of results | 14 | Describe the methods of handling data and combining results of studies, if done, including measures of consistency (e.g., I^2^) for each meta-analysis. | | n.a. |  |
| **Section/topic** | **#** | | **Checklist item** | **Reported on page #** | |
| Risk of bias across studies | 15 | | Specify any assessment of risk of bias that may affect the cumulative evidence (e.g., publication bias, selective reporting within studies). | 4 | |
| Additional analyses | 16 | | Describe methods of additional analyses (e.g., sensitivity or subgroup analyses, meta-regression), if done, indicating which were pre-specified. | n.a. | |
| **RESULTS** | | | |  | |
| Study selection | 17 | | Give numbers of studies screened, assessed for eligibility, and included in the review, with reasons for exclusions at each stage, ideally with a flow diagram. | 4 and Fig. 1 | |
| Study characteristics | 18 | | For each study, present characteristics for which data were extracted (e.g., study size, PICOS, follow-up period) and provide the citations. | 4, 5 and Table 1, 2 | |
| Risk of bias within studies | 19 | | Present data on risk of bias of each study and, if available, any outcome level assessment (see item 12). | 4, 5 and Fig. 2 | |
| Results of individual studies | 20 | | For all outcomes considered (benefits or harms), present, for each study: (a) simple summary data for each intervention group (b) effect estimates and confidence intervals, ideally with a forest plot. | Table 1, 2 | |
| Synthesis of results | 21 | | Present results of each meta-analysis done, including confidence intervals and measures of consistency. | n.a. | |
| Risk of bias across studies | 22 | | Present results of any assessment of risk of bias across studies (see Item 15). | 4, 5 and Fig. 2 | |
| Additional analysis | 23 | | Give results of additional analyses, if done (e.g., sensitivity or subgroup analyses, meta-regression [see Item 16]). | n.a. | |
| **DISCUSSION** | | | |  | |
| Summary of evidence | 24 | | Summarize the main findings including the strength of evidence for each main outcome; consider their relevance to key groups (e.g., healthcare providers, users, and policy makers). | 5-7 | |
| Limitations | 25 | | Discuss limitations at study and outcome level (e.g., risk of bias), and at review-level (e.g., incomplete retrieval of identified research, reporting bias). | 7 | |
| Conclusions | 26 | | Provide a general interpretation of the results in the context of other evidence, and implications for future research. | 8 | |
| **FUNDING** | | | |  | |
| Funding | 27 | | Describe sources of funding for the systematic review and other support (e.g., supply of data); role of funders for the systematic review. | 8 | |

# Supplementary Table 2. The Quality in Prognostic Studies (QUIPS) tool.

| Domains | Criteria |
| --- | --- |
| Study participation | 1. Adequate participation in the study by eligible persons 2. Description of the source population or population of interest 3. Description of the baseline study sample 4. Adequate description of the sampling frame and recruitment 5. Adequate description of the period and place of recruitment 6. Adequate description of inclusion and exclusion criteria |
| Study attrition | 1. Adequate response rate for study participants 2. Description of attempts to collect information on participants who dropped out 3. Reasons for loss to follow-up are provided 4. Adequate description of participants lost to follow-up 5. There are no important differences between participants who completed the study and those who did not |
| Prognostic factor measurement | 1. A clear definition or description of the prognostic factor is provided 2. Method of prognostic factor measurement is adequately valid and reliable 3. Continuous variables are reported or appropriate cut points are used 4. The method and setting of measurement of prognostic factor is the same for all study participants 5. Adequate proportion of the study sample has complete data for the prognostic factor 6. Appropriate methods of imputation are used for missing prognostic factor data |
| Outcome measurement | 1. A clear definition of the outcome is provided 2. Method of outcome measurement used is adequately valid and reliable 3. The method and setting of outcome measurement is the same for all study participants |
| Other prognostic factors (covariates) | 1. Other prognostic factors (covariates) are measured 2. Clear definitions of the important prognostic factors (covariates) measured are provided 3. Measurement of all important prognostic factors (covariates) is adequately valid and reliable 4. The method and setting of prognostic factor measurement are the same for all study participants 5. Appropriate methods are used if imputation is used for missing data 6. Important potential prognostic factors (covariates) are accounted for in the study design 7. Important potential prognostic factors (covariates) are accounted for in the analysis |
| Statistical analysis and reporting | 1. Sufficient presentation of data to assess the adequacy of the analytic strategy 2. Strategy for model building is appropriate and is based on a conceptual framework or model 3. The selected statistical model is adequate for the design of the study 4. There is no selective reporting of results |

Four judgements:

1. Low risk of bias: the relationship between the prognostic factor and outcome is unlikely to be different for participants and eligible non-participants.
2. Moderate risk of bias: the relationship between the prognostic factor and outcome may be different for participants and eligible non-participants.
3. High risk of bias: the relationship between the prognostic factor and outcome is very likely to be different for participants and eligible non-participants.
4. Unclear risk of bias: the study does not provide sufficient information that allows a clear judgement for this domain
